# Evaluating a Multi-Camera Markerless System for Capturing Basketball-Specific Movements: An Exploration Using 25 Hz Video Streams

**DOI:** 10.3390/s26051689

**Published:** 2026-03-07

**Authors:** Zhaoyu Li, Zhenbin Tan, Wen Zheng, Ganling Yang, Junye Tao, Mingxin Zhang, Xiao Xu

**Affiliations:** 1 Institute of Physical Education and Training, Capital University of Physical Education and Sports, Beijing 100191, China; lizhaoyu2022@cupes.edu.cn (Z.L.); tanzhenbin@cupes.edu.cn (Z.T.); 2 School of Athletic Performance, Shanghai University of Sport, Shanghai 200438, China; 2321852032@sus.edu.cn (W.Z.); 2521811001@sus.edu.cn (G.Y.); 3 College of Physical Education and Sports, Beijing Normal University, Beijing 100875, China; 202411070032@mail.bnu.edu.cn; 4 College of Physical Education, Dalian University, Dalian 116622, China

**Keywords:** basketball, markerless motion capture, kinematic validation, deep learning

## Abstract

Markerless motion capture (MMC) provides a non-invasive alternative for motion analysis; however, its validity at the standard frame rate of 25 Hz commonly used in broadcast and surveillance applications remains to be established. This study evaluated the performance of a 25 Hz multi-camera MMC workflow using consumer-grade cameras for capturing basketball-specific movements. Three highly trained male athletes completed seven tasks, including sprinting and simulated sport-specific skills, while being synchronously recorded by six MMC cameras (DJI Action 5 Pro, 25 fps) and a 10-camera Vicon system (25 Hz). Kinematic data were processed using an RTMDet–RTMPose pipeline and low-pass filtered at 6 Hz. Waveform validity was assessed using Pearson’s correlation coefficient (r) and the root mean square error (RMSE). The displacement magnitudes of 12 joints showed excellent agreement (r = 0.916–0.994; median nRMSE = 0.54–1.32%), indicating robust trajectory reconstruction. In contrast, agreement decreased for derivative variables: velocity (r = 0.583–0.867) and acceleration (r = 0.232–0.677) were highly sensitive to the low sampling rate and numerical differentiation. Although a 25 Hz configuration is insufficient for high-precision impact analysis, it provides acceptable accuracy for macroscopic displacement tracking and external-load quantification in resource-constrained training environments. Future optimization should prioritize temporal synchronization to improve the reliability of derivative variables.

## 1. Introduction

Basketball is characterized by rapid accelerations and decelerations, frequent changes of direction, explosive jump–landing cycles, and highly coordinated upper- and lower-limb actions [1]. Quantifying three-dimensional (3D) joint kinematics during these sport-specific actions is essential for evidence-based training, injury-risk screening, return-to-play decision-making, and long-term performance monitoring [2,3]. In recent years, optical marker-based motion-capture systems (e.g., Vicon) have been widely accepted as a reference method for 3D kinematics because of their high spatial accuracy and mature processing workflows [4]. However, marker-based systems remain difficult to deploy in real-world sport settings, as they require dedicated infrastructure, time-consuming marker placement, and a limited capture volume that can be exceeded by fast, large-displacement training actions [5,6]. In addition, markers may detach, become occluded, or introduce soft-tissue artifacts, all of which can bias kinematic outputs, particularly during high-speed movements [7].

Recent advances in deep-learning-based pose estimation have accelerated the development of markerless motion capture (MMC) systems [8,9]. These approaches identify anatomical keypoints directly from video and reconstruct human motion trajectories, offering non-invasive, low-barrier, and scalable measurement solutions [9,10,11]. Importantly, multi-camera MMC provides redundancy to mitigate partial occlusion and can preserve trajectory continuity even when athletes move near the boundary of the capture volume [12]. Compared with Vicon [4], it is particularly suitable for continuous acquisition and analysis of natural movement patterns in training environments [13,14]. Prior studies have shown that MMC can achieve accuracy comparable to marker-based systems for lower-limb joint kinematics and selected kinetic outcomes. Across multi-task, non-sport-specific settings (e.g., running, jumping, squatting, and cutting), MMC has generally demonstrated acceptable accuracy and reliability for kinematic assessment of the lower limbs, trunk, and upper limbs [4,15,16,17]. Nevertheless, most validation studies rely on expensive high-frame-rate industrial cameras (>60 Hz, and often 100 Hz), which constrain broader adoption in large-scale sport programs and resource-limited settings. In practice, 25 or 30 Hz video streams remain the most common data source in game broadcasts, routine training recordings, and standard venue surveillance systems.

Despite these advances, several challenges must be addressed before multi-camera MMC can be considered a reliable alternative for sport-specific biomechanical measurement. First, most validation work has focused on walking or generic functional tasks, whereas basketball imposes more complex demands on coordination, limb speed, and self-occlusion [9,12]. Second, agreement in positional signals does not necessarily translate to accurate derivatives such as velocity and acceleration. This is particularly critical at low frame rates, where numerical differentiation amplifies small spatial noise and attenuates high-frequency impact-related content [18]. Accordingly, defining the performance boundaries of low-frame-rate MMC—between macroscopic external-load metrics (e.g., displacement and mean velocity) and microscopic kinematic features (e.g., instantaneous velocity)—is crucial for real-world deployment. Third, beyond cross-system validity, practical applications require test–retest reliability, because the usefulness of MMC for training monitoring depends on whether observed changes exceed measurement noise [19,20].

Therefore, this study evaluated the concurrent validity and test–retest reliability of a consumer-grade 25 Hz multi-camera markerless motion capture (MMC) workflow for basketball-specific movements, using a laboratory-grade 10-camera Vicon system as the reference standard. The primary research question was: to what extent does this low-frame-rate MMC approach agree with Vicon when quantifying 3D joint displacement, velocity, and acceleration across major upper- and lower-limb joints during representative basketball tasks? We hypothesized that MMC would show excellent waveform agreement for displacement across joints and tasks (r > 0.90), whereas agreement for velocity and acceleration would be substantially lower. In addition, we assessed test–retest reliability across two testing days to determine whether trial-level MMC metrics are sufficiently repeatable for practical training monitoring applications.

## 2. Materials and Methods

### 2.1. Participants

Three experienced male basketball athletes were recruited (age: 25.00 ± 4.58 years; height: 189.33 ± 4.04 cm; body mass: 91.00 ± 17.69 kg; training experience: 7.00 ± 1.00 years). Inclusion criteria were: (i) ability to proficiently perform all experimental tasks; (ii) no history of severe lower-limb injury within the previous two years; and (iii) absence of acute conditions during testing that could affect performance. All participants provided written informed consent. The protocol was approved by the Scientific Research Ethics Committee of Shanghai University of Sport.

### 2.2. Experimental Setup and Data Acquisition

**Figure 1:**
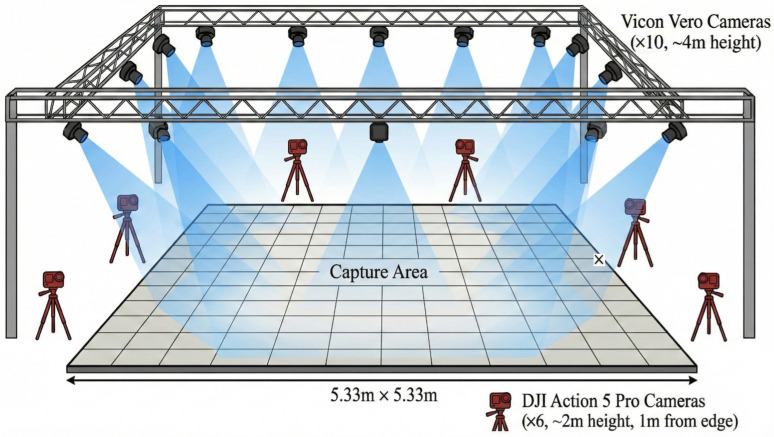
Experimental site for the dual-system synchronized validation.

Experiments were conducted in the Biomechanics Laboratory of Shanghai University of Sport. The overall experimental capture setup is shown in Figure 1. The reference system was a 10-camera Vicon optical motion-capture setup (Vicon Vero) surrounding an approximately 5.33 m × 5.33 m capture area (camera height ~4 m), recorded in Vicon Nexus. This configuration represents a standard laboratory reference setup with high camera redundancy to minimize reconstruction uncertainty. The MMC system used six consumer-grade action cameras (DJI Action 5 Pro, SZ DJI Technology Co., Ltd., Shenzhen, China) positioned around the same capture area (approximately 1 m outside the boundary; height ~2 m), recording at 1920 × 1080 resolution and 25 fps. Both systems were intentionally configured at the same nominal sampling rate (25 Hz) to enable a like-for-like comparison. The MMC camera count was deliberately limited to six to reflect realistic deployment constraints for 3 × 3 basketball training/competition monitoring, where space, budget, setup time, and interference with play make larger portable arrays difficult; consequently, lower redundancy may reduce keypoint visibility and triangulation robustness during partially occluded, high-speed movements [21]. Camera calibration followed a fixed-camera, moving-checkerboard multi-view procedure; intrinsic and extrinsic parameters were estimated to define a unified world coordinate system. Calibration quality was assessed using mean reprojection error (~2 pixels), which was deemed acceptable for subsequent triangulation.

### 2.3. MMC Pipeline and Marker Configuration

To obtain 3D joint trajectories, we implemented a top-down multi-view 3D reconstruction workflow. Multi-view videos were first temporally synchronized using Mel-frequency cepstral coefficients (MFCC) extracted from audio signals to achieve frame-level alignment across views [22]. For each view, person detection was performed using RTMDet, followed by RTMPose to output COCO-style 2D keypoint coordinates and confidence scores [23,24]. The MMC 2D pose estimation employed the official RTMPose model (OpenMMLab MMPose framework) with COCO-pretrained weights further trained on general human pose datasets, without basketball-specific fine-tuning. This generic, out-of-the-box configuration was deliberately chosen to evaluate the realistic performance boundary of a consumer-grade pipeline readily deployable by coaches and researchers without domain-specific labelled data. Compared with earlier models, RTMPose has demonstrated improved pose-estimation accuracy in prior work [25].

For trajectory tracking, we used the OCSORT tracker combined with SOLIDER re-identification, and introduced a 3D-matching penalty during association to improve robustness [26,27]. After obtaining 2D pose trajectories for each view, 3D pose sequences were reconstructed using triangulation [28]. The MMC model outputs 17 COCO body keypoints (nose; left/right eyes; left/right ears; left/right shoulders; left/right elbows; left/right wrists; left/right hips; left/right knees; left/right ankles). For cross-system comparison, we selected 12 limb-related keypoints (shoulders, elbows, wrists, hips, knees, and ankles; left/right) that have direct anatomical relevance to our basketball-specific movements and can be matched to Vicon markers or marker-derived joint centers. The 5 facial keypoints (nose, eyes, and ears) were excluded because they have no corresponding reflective markers in our Vicon setup, are prone to occlusion and less stable detection in sport-like trials, and are not required for the joint-level kinematic comparisons targeted in this study.

**Figure 2:**
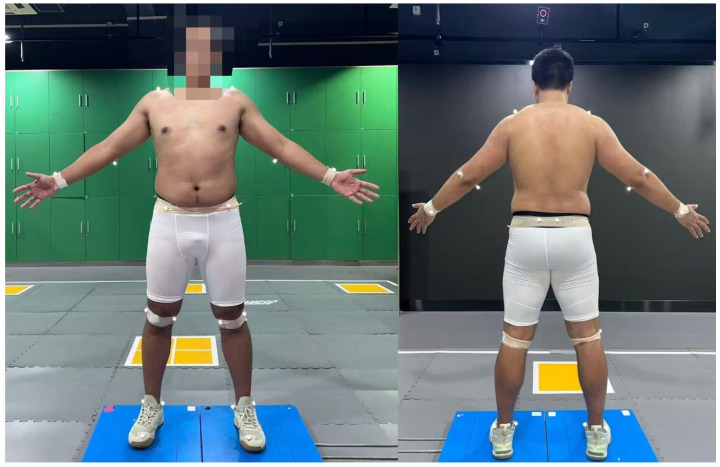
Schematic of marker placement.

Vicon marker placement followed a modified anatomical-landmark protocol commonly used in optical motion capture [29,30], with minor adaptations to enhance anatomical comparability with the selected MMC COCO keypoints and to improve the stability of marker-derived joint-center estimation. The marker placement configuration and participant standing posture are illustrated in Figure 2. Specifically, 22 reflective markers used for cross-system kinematic analyses were placed on major anatomical landmarks: bilateral shoulders (acromion region; 2), elbows (medial and lateral epicondyles; 4), wrists (radial and ulnar styloid processes; 4), hips/pelvis (bony prominences near the anterior and posterior superior iliac spines; 4), knees (medial and lateral femoral condyles; 4), and ankles (medial and lateral malleoli; 4). In addition, two shoe-mounted markers located near the toe region were used solely to facilitate take-off event identification for cross-system temporal alignment (MMC vs. Vicon); these markers were not included in any subsequent trajectory-based computations, kinematic derivations, or cross-system comparisons.

Accordingly, our primary validation targets were kinematic quantities derived from these joint trajectories—displacement, velocity, and acceleration—because they are directly obtainable from both systems and are most relevant to practical training/competition monitoring. In particular, magnitude-based kinematics are less sensitive to constant spatial offsets that may arise from different anatomical definitions, while numerical differentiation still reflects the real-world propagation of micro-errors into first- and second-order derivatives. By contrast, estimating whole-body center of mass would require additional biomechanical modeling assumptions, which could confound an “out-of-the-box” system-to-system comparison. Center-of-mass metrics can be derived post hoc from the same 3D trajectories and will be explored in future work.

### 2.4. Task Protocol and Synchronization

Before each testing session, participants completed a ~10 min standardized warm-up (3 min light jogging, 4 min dynamic stretching, and three sets of low-intensity lateral shuffles and small hops) to ensure adequate range of motion and neuromuscular activation. Participants then stood naturally at the center of the capture area for ~3 s to check marker visibility and static posture quality. Prior to data collection, the Vicon system was calibrated following the standard procedure, including camera calibration and wand/plate-based volume definition. An operator moved a T-shaped calibration wand throughout the capture volume with wide-range, multi-directional motions to ensure visibility across cameras, and then placed the wand flat on the floor at the center of the testing area to complete calibration. Reflective markers were placed on four floor reference landmarks, and their 3D coordinates were recorded to establish a unified world coordinate system for that session.

**Figure 3:**
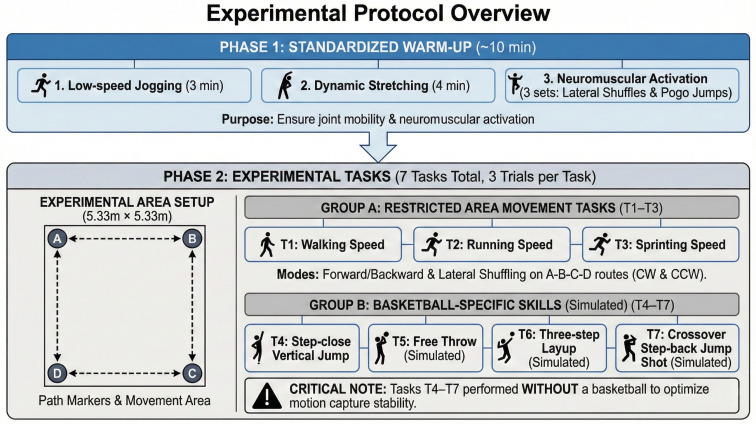
Schematic of the experimental procedure.

To synchronize Vicon and MMC, before each task trial the researcher gave a verbal cue and the participant performed a single vertical jump in place, then stood still before initiating the task. This event was clearly identifiable in the multi-view videos and produced an abrupt change in Vicon marker trajectories; the corresponding frame was used as the synchronization reference (t_sync_) to align both systems at the frame level. At least 1 min rest was provided between high-intensity tasks and extended when necessary based on perceived fatigue to minimize fatigue-related degradation in movement quality and trajectory stability. Seven task conditions were tested (see Figure 3 for an overview of the experimental procedure and task set): three key-area locomotion conditions (walking/running/sprinting), a step-in vertical jump, a free-throw posture simulation, a three-step layup, and a crossover step-back jump shot. For the key-area locomotion task, the four corners of the capture area (A, B, C, D) served as path markers; participants performed shuttle movements along the prescribed route at walking, running, and sprinting speeds. To reduce interference from the ball on keypoint detection and marker visibility, all three basketball skill tasks were performed without a ball (simulated movements). This choice minimized occlusion and improved tracking stability for cross-system validation; ball-present conditions will be examined in future work. Each participant performed one trial per task on each testing day. Across two days, this yielded 3 (participants) × 2 (days) × 7 (tasks) = 42 paired synchronized trials for analysis.

### 2.5. Data Processing and Outcome Variables

Vicon and MMC outputs were exported as time series of 3D joint-center coordinates in the world coordinate system, with the same sampling frequency (25 Hz). The two systems were time-aligned using the synchronization event (a vertical jump performed immediately before each trial). After synchronization, paired trials were merged frame-by-frame using a shared frame index, and only the overlapping frame range present in both systems was retained for subsequent analyses. To reduce the influence of between-trial initial position offsets, joint coordinates were converted to relative displacements with respect to the first retained frame. For each joint, raw coordinates were denoted as *X*(*t*), *Y*(*t*), and *Z*(*t*), and relative displacement components were computed as follows:

(1) $$x\left(t\right)=X\left(t\right)-X\left(t_{0}\right),\;y\left(t\right)=Y\left(t\right)-Y\left(t_{0}\right),\;z(t)=Z(t)-Z(t_{0})$$

Here, $t_{0}$ denotes the first frame of the overlapped segment after frame-wise merging. Each component signal was then filtered using a 4th-order low-pass Butterworth filter with a 6 Hz cutoff, implemented with zero-phase forward–backward filtering (filtfilt) to avoid phase delay [31]. Filtering was implemented in Python 3.10 using scipy.signal.filtfilt. Given the 25 Hz sampling rate and the amplification of high-frequency noise in derivative signals, a conservative 6 Hz cutoff was selected to suppress 2D/3D reconstruction jitter and differentiation noise in velocity/acceleration estimates, aligning with the intended use of consumer devices in resource-limited settings. Velocity and acceleration were computed via numerical differentiation of the filtered displacement signals. The sampling interval was $\Delta t\;=\frac{1}{25}$ S, and the first derivative was estimated using a central-difference scheme. A central-difference scheme was chosen because it is symmetric (time-centered) and second-order accurate, which reduces numerical bias compared with one-sided differences when estimating derivatives from discretely sampled kinematic time series [32].

(2) $$v_{x}\left(t\right)=\frac{x\left(t+1\right)-x\left(t-1\right)}{2\Delta t},\;v_{y}\left(t\right)=\frac{y\left(t+1\right)-y\left(t-1\right)}{2\Delta t},\;v_{z}\left(t\right)=\frac{z\left(t+1\right)-z\left(t-1\right)}{2\Delta t}$$

Similarly, acceleration components were computed using second-order central differences.

(3) $$a_{x}\left(t\right)=\frac{x\left(t+1\right)-2x\left(t\right)+x\left(t-1\right)}{{\left(\Delta t\right)}^{2}},\;a_{y}\left(t\right)=\frac{y\left(t+1\right)-2y\left(t\right)+y\left(t-1\right)}{{\left(\Delta t\right)}^{2}},a_{z}\left(t\right)=\;\frac{z\left(t+1\right)-2z\left(t\right)+z\left(t-1\right)}{{\left(\Delta t\right)}^{2}}$$

Because central differences require neighboring frames, the first and last frames of each trial were excluded from derivative-based analyses. In addition to axis-specific components, magnitude (vector-norm) variables were calculated to provide direction-independent summaries of movement intensity.

(4) $${pos}_{mag}\left(t\right)=\sqrt{x{\left(t\right)}^{2}+y{\left(t\right)}^{2}+z{\left(t\right)}^{2}}$$

(5) $$v_{mag}\left(t\right)=\sqrt{v_{x}{\left(t\right)}^{2}+v_{y}{\left(t\right)}^{2}+v_{z}{\left(t\right)}^{2}}$$

(6) $$a_{mag}\left(t\right)=\sqrt{a_{x}{\left(t\right)}^{2}+a_{y}{\left(t\right)}^{2}+a_{z}{\left(t\right)}^{2}}$$

These magnitude variables—displacement magnitude (pos_mag_), velocity magnitude (v_mag_), and acceleration magnitude (a_mag_)—served as primary outcomes for waveform validity/agreement and inter-system agreement analyses. For reliability analyses, trial-level features were extracted from each magnitude time series, including the mean, peak, and 95th percentile (P95).

### 2.6. Statistical Analysis

All data processing and statistical analyses were performed in Python 3.10 (NumPy, pandas and SciPy). After frame-level alignment and 6 Hz low-pass filtering, displacement magnitude (${pos}_{mag}$), velocity magnitude ($v_{mag}$), and acceleration magnitude ($a_{mag}$) were computed for 12 joints, using paired trials as the statistical unit [33]. Waveform validity/agreement was assessed using Pearson’s correlation coefficient (r) to quantify shape similarity, and RMSE and nRMSE to quantify amplitude error. nRMSE% was normalized by the full-sample amplitude range (max–min) of the corresponding Vicon waveform for the same joint and variable and expressed as a percentage. Because nRMSE% can be inflated when the reference range is small (e.g., near-static segments), it was interpreted alongside RMSE. Inter-system agreement was evaluated using Bland–Altman analysis, reporting mean bias and 95% limits of agreement (LoA = bias ± 1.96 × SD) [34]. To avoid treating frame-level observations as independent samples, repeated-measures Bland–Altman methods were applied to obtain more robust LoA estimates. Lin’s concordance correlation coefficient (CCC) was reported as a complementary agreement metric [35]. Test–retest reliability across two testing days was assessed using trial-level features (mean, peak, P95) extracted from the magnitude series. Reliability was quantified using the ICC (two-way random-effects, absolute agreement, single measurement; ICC(A,1)) with 95% confidence intervals, and absolute error was summarized using CV%, SEM, and MDC95 ($MDC95\;=\;1.96\;\times \;\sqrt{2}\;\times \;SEM$) [36,37]. For reporting, r and RMSE are presented as mean ± SD, whereas nRMSE% is presented as median [IQR] to reduce denominator effects in cross-condition comparisons. The main text reports test–retest reliability results for the mean feature only, whereas reliability results for peak and P95 are provided in the Supplementary Materials. The same filtering and the same numerical differentiation scheme were applied to both MMC and Vicon signals to ensure a like-for-like comparison. During pipeline development, alternative cutoff settings were evaluated, and a conservative 6 Hz cutoff was adopted to suppress frame-to-frame jitter prior to numerical differentiation.

## 3. Results

### 3.1. Data Quality Control and Paired-Trial Overview

At the trial level, MMC and Vicon data were successfully paired in a 1:1 manner, yielding 42 paired trials included for subsequent analyses. All paired trials were frame-aligned and cropped to the overlapping segment using a shared frame_index_. Data integrity checks identified one paired trial in which the Vicon right-shoulder trajectory was missing the *z*-axis component. Accordingly, for trial-level waveform validity analyses (r/RMSE/nRMSE%), this issue affected only the right-shoulder statistics (right-shoulder: *n* = 41; all other joints: *n* = 42). For frame-level agreement analyses requiring complete 12-joint vectors (pooled Bland–Altman and CCC), the affected paired trial was excluded a priori based on predefined criteria; thus, frame-level analyses were conducted on 41 paired trials.

### 3.2. Overall Waveform Validity Across 12 Joints

Table 1, Table 2 and Table 3 summarize the overall waveform validity between MMC and Vicon across 12 joints for three kinematic magnitude variables: displacement magnitude (pos_mag_), velocity magnitude (v_mag_), and acceleration magnitude (a_mag_). Waveform shape similarity was quantified using Pearson’s correlation coefficient (*r*), whereas amplitude error was quantified using RMSE and normalized RMSE (nRMSE%, reported as median [IQR]).

As shown in Table 1, displacement magnitude (pos_mag_) demonstrated high waveform agreement across joints (r_mean_ = 0.916–0.994) with small amplitude errors (RMSE_mean_ = 0.075–0.176 m; median nRMSE% = 0.54–1.32%). In Table 2, waveform agreement for velocity magnitude (v_mag_) decreased relative to displacement (r_mean_ = 0.583–0.867), accompanied by larger errors (RMSE_mean_ = 0.670–1.285 m/s; median nRMSE% = 0.62–3.76%). Table 3 further indicates that acceleration magnitude (a_mag_) exhibited the lowest agreement (r_mean_ = 0.232–0.677) and markedly higher RMSE values (14.294–31.906 m/s^2^; median nRMSE% = 0.30–8.45%). Overall, waveform validity followed a consistent pattern across variables, with posmag outperforming v_mag_, and v_mag_ outperforming a_mag_. Joint-wise distributions of waveform correlations are shown in the Supplementary Materials (Figure S6).

### 3.3. Task-Stratified Waveform Validity Across Seven Movement Tasks

To compare task-dependent differences in waveform agreement between MMC and Vicon, trial-level validity metrics were stratified and summarized by task condition. Table 4 reports, for each of the seven tasks, the across-joint mean correlation coefficient ($r_{mean}$) and mean RMSE (${RMSE}_{mean}$) for three magnitude variables (pos_mag_, v_mag_, and a_mag_). Because one trial in Tri01 had a missing right-shoulder field, the number of observations was *n* = 71 for Tri01, whereas all other tasks had *n* = 72.

For displacement magnitude (pos_mag_), all tasks showed consistently high agreement ($r_{mean}$ = 0.939–0.999). Key-area locomotion (Tri01–Tri03) and the step-in vertical jump (Tri04) approached ceiling-level correlations ($r_{mean}$ = 0.996–0.999) with small errors (${RMSE}_{mean}$ = 0.029–0.086 m). Among the basketball skill tasks, the free-throw simulation (Tri05) exhibited the lowest displacement agreement ($r_{mean}$ = 0.939, ${RMSE}_{mean}$ = 0.045 m). The three-step layup (Tri06) and crossover step-back jump shot (Tri07) maintained high correlations ($r_{mean}$ = 0.972–0.978), but with larger errors (Tri06: ${RMSE}_{mean}$ = 0.366 m; Tri07: ${RMSE}_{mean}$ = 0.124 m).

For velocity magnitude (v_mag_), between-task variability increased markedly ($r_{mean}$ = 0.181–0.866). Key-area locomotion and the step-in vertical jump (Tri01–Tri04) showed moderate-to-high agreement ($r_{mean}$ = 0.778–0.824) with ${RMSE}_{mean}$ = 0.294–0.704 m/s, whereas the free-throw simulation (Tri05) yielded the highest velocity agreement ($r_{mean}$ = 0.866, ${RMSE}_{mean}$ = 0.199 m/s). In contrast, the three-step layup (Tri06) showed the lowest velocity agreement and the largest error ($r_{mean}$ = 0.181, ${RMSE}_{mean}$ = 4.423 m/s).

For acceleration magnitude (a_mag_), task dependence became even more pronounced ($r_{mean}$ = 0.061–0.595). Key-area locomotion tasks showed relatively low correlations (Tri01–Tri03: $r_{mean}$ = 0.402–0.434), and the sprinting condition exhibited higher error (Tri03: ${RMSE}_{mean}$ = 17.902 m/s^2^). The step-in vertical jump and free-throw simulation (Tri04–Tri05) showed higher correlations ($r_{mean}$ = 0.574–0.593) with ${RMSE}_{mean}$ = 5.228–10.321 m/s^2^, and the crossover step-back jump shot (Tri07) also performed relatively well ($r_{mean}$ = 0.595, ${RMSE}_{mean}$ = 6.002 m/s^2^). The three-step layup (Tri06) exhibited the lowest acceleration agreement and the largest error ($r_{mean}$ = 0.061, ${RMSE}_{mean}$ = 102.351 m/s^2^). Records of the anomalous trials in Tri06, supplementary visualizations, and sensitivity analyses are provided in the Supplementary Materials.

### 3.4. Waveform Visualization

**Figure 4:**
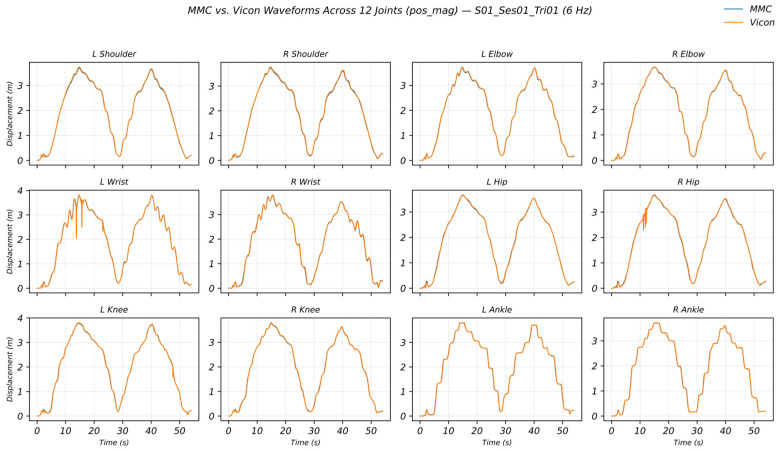
MMC vs. Vicon waveforms across 12 joints for displacement magnitude (pos_mag_) in a representative trial. Vicon is shown in orange and MMC in blue (both solid lines).

Figure 4 presents a time-series comparison of displacement magnitude (pos_mag_) waveforms across 12 joints in a representative paired trial. Overall, MMC and Vicon showed highly consistent peak–trough timing and overall waveform trends; for a few joints, local amplitude deviations or slight temporal offsets were observed during rapidly changing segments. Representative waveform comparisons for velocity and acceleration magnitudes (v_mag_ and a_mag_) are provided in the Supplementary Materials (Figures S1 and S2).

### 3.5. Frame-Level Agreement Between MMC and Vicon

To evaluate frame-level agreement, pooled Bland–Altman plots were used for visualization, and repeated-measures Bland–Altman analyses were performed to estimate the overall bias and 95% limits of agreement (LoA). Because one paired trial contained incomplete right-shoulder 3-axis data, this trial was excluded from frame-level analyses, leaving 41 valid paired trials. The resulting numbers of pooled “joint × frame” observations were 510,540 for pos_mag_, 510,048 for v_mag_, and 509,556 for a_mag_ (Table 5). To facilitate interpretation of the frame-level agreement results, it should be noted that each point in the pooled Bland–Altman plot represents a paired MMC–Vicon observation at a specific frame for a specific joint (i.e., a joint–frame pair). The mean difference (bias) reflects the systematic offset between systems, whereas the 95% limits of agreement (LoA = bias ± 1.96 × SD) quantify the expected range within which most frame-level differences fall. Because frames are nested within trials and subjects (i.e., repeated measurements), treating all frames as independent would underestimate variability. Therefore, we report repeated-measures Bland–Altman estimates, which provide more robust LoA by accounting for within-trial/within-subject dependence.

**Figure 5:**
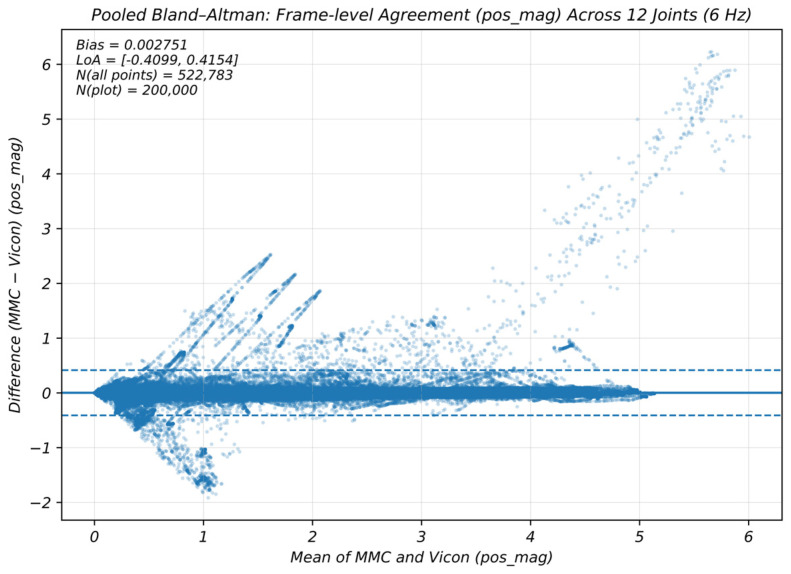
Pooled Bland–Altman plot of frame-level agreement between MMC and Vicon for displacement magnitude (pos_mag_) across 12 joints.

As shown in Figure 5, the pos_mag_ differences were centered around zero with relatively small dispersion. Table 5 indicates that the pos_mag_ bias was close to zero (bias = 0.0029 m), with LoA ranging from −0.4252 to 0.4310 m. In contrast, differences for v_mag_ and a_mag_ exhibited greater dispersion. Table 5 shows slight negative biases for v_mag_ and a_mag_ (bias = −0.0163 m/s and −1.0451 m/s^2^, respectively), accompanied by wider LoA. Results for Lin’s concordance correlation coefficient (CCC) are reported in the Supplementary Materials. The corresponding pooled Bland–Altman plots for v_mag_ and a_mag_ are provided in the Supplementary Materials (Figures S3 and S4).

### 3.6. Test–Retest Reliability and Measurement Error

Test–retest reliability and measurement error were further evaluated across two testing days. For each trial, mean, peak, and 95th percentile (P95) features were extracted from the pos_mag_, v_mag_, and a_mag_ magnitude series, and ICC(A,1), CV%, SEM, and MDC95 were computed. To limit manuscript length, only the mean-feature results are summarized in the main text (Table 6), whereas results for peak and P95 are provided in the Supplementary Materials (Tables S3 and S4).

Table 6 suggests that day-to-day stability for the mean feature was broadly comparable between the two systems. The median CV% values for MMC were 3.97% (pos_mag_), 3.93% (v_mag_), and 6.22% (a_mag_), while the corresponding values for Vicon were 4.00%, 4.52%, and 12.99%, respectively. The median MDC95 values were 0.111 (pos_mag_), 0.073 (v_mag_), and 0.504 (a_mag_) for MMC, and 0.113, 0.075, and 0.787 for Vicon, respectively (Table 6). ICC(A,1) estimates are also reported in Table 6.

## 4. Discussion

This study evaluated the 3D kinematic performance of a markerless motion capture (MMC) approach based on deep-learning pose estimation and multi-view 3D reconstruction for basketball-specific movements, using a laboratory-grade optical motion capture system (Vicon) as the reference. Overall, MMC stably reproduced the major spatiotemporal patterns of joint trajectories at the displacement-magnitude level (pos_mag_): waveform correlations across 12 joints remained high (r = 0.916–0.994), with relatively small displacement errors (RMSE = 0.075–0.176 m). Agreement analyses further supported this finding: repeated-measures Bland–Altman results showed a negligible displacement bias (bias ≈ 0.0029 m) with limits of agreement of approximately −0.4252 to 0.4310 m. These findings suggest that, without reflective markers or complex instrumentation, a multi-camera video–based MMC workflow is practically feasible for training monitoring and movement assessment at the level of displacement trajectory reconstruction and other low-order kinematic features, consistent with prior markerless validation studies reporting high agreement for displacement-related measures [38,39]. Importantly, this displacement-dominant agreement pattern is also aligned with broader markerless validation evidence in functional and sport-like tasks, where position/displacement signals tend to be substantially more robust than numerically differentiated variables [31].

To mitigate the large discrepancies in derivative variables, we focused on suppressing high-frequency jitter before numerical differentiation. In particular, we evaluated alternative low-pass cutoff settings during development and adopted a conservative 6 Hz, zero-phase filter for both systems to improve derivative interpretability, while acknowledging that extreme transients are attenuated under this setting. We did not switch pose networks or perform basketball-specific fine-tuning in the present study because our aim was to benchmark an out-of-the-box MMC workflow, and the dataset did not provide task-specific labeled 2D keypoints or 3D ground truth for supervised adaptation. Nevertheless, domain-specific fine-tuning on basketball imagery (with 2D labels) may further reduce keypoint jitter and could be a practical route to improving velocity and acceleration agreement in future work.

Importantly, agreement decreased as the kinematic order increased, which is attributable to the amplification of subtle temporal offsets and high-frequency jitter during numerical differentiation. Compared with displacement, derivative variables showed reduced agreement: joint-level correlations for velocity magnitude (v_mag_) were r = 0.583–0.867 with larger errors (RMSE = 0.670–1.285 m/s), while acceleration magnitude (a_mag_) exhibited greater joint-dependent variability (r = 0.232–0.677; RMSE = 14.294–31.906 m/s^2^). This monotonic degradation from displacement to velocity and then acceleration reflects both aliasing under low sampling rates and noise amplification inherent to numerical differentiation [40]. Under the 6 Hz filtering setting used here, the system captured the macroscopic movement trends but inevitably smoothed high-frequency transient features common in basketball, such as take-off and abrupt deceleration [41]. This observation is consistent with established motion-capture considerations: even when displacement reconstruction is highly consistent, small timing offsets or differences in inherent smoothing strategies can produce substantial deviations in derivative waveforms [42]. Similar conclusions have been reported in other markerless validation efforts, where position-level tracking was generally acceptable but derivative- or high-dynamic outcomes were more error-prone, especially under rapid motions and partial occlusions [43,44]. Accordingly, derivative variables from the 25 Hz MMC workflow are more appropriate for relative, low-frequency/aggregate intensity descriptors (e.g., trial-level mean or percentile-based metrics such as P95) rather than for quantifying instantaneous peak values in absolute terms.

At the task level, MMC yielded higher derivative agreement in relatively controlled movements with minimal occlusion (e.g., free-throw simulation), whereas discrepancies became more pronounced in tasks involving larger displacements, sharper tempo changes, or more complex postural dynamics. For example, in the three-step layup (Tri06), correlations for velocity and acceleration were markedly lower and RMSE values were substantially larger, indicating the weakest inter-system agreement for derivative variables under this task condition. This pattern may be attributable to the task’s rapid translation and posture changes and may also be jointly influenced by capture-volume boundaries, occlusion, and 3D reconstruction stability. In addition, the lower camera redundancy of the six-camera markerless setup may have further amplified these effects during high-speed actions by reducing multi-view keypoint visibility and triangulation robustness when occlusions occur; therefore, the observed task-dependent discrepancies should be interpreted as the result of multiple interacting factors, rather than being attributed to any single cause.

From a joint-specific perspective, proximal joints (e.g., shoulders and hips) generally exhibited higher displacement-level agreement, whereas distal joints—particularly the wrist—were more challenging, likely due to the sensitivity of vision-based tracking to small, highly dynamic segments and the higher probability of occlusion for distal joints during sport-specific actions [44,45]. In addition, COCO keypoint definitions used by MMC are not anatomically identical to joint centers inferred by Vicon from the geometric centers of reflective markers. Such model-definition differences are a known source of systematic offsets; even with highly consistent waveform trends, they can lead to larger dispersion in the magnitude of distal segments [38]. Nevertheless, the mean displacement correlation for the wrist remained above 0.90, indicating that the system can still provide practically useful displacement-trajectory information for most basketball-specific movements.

Regarding test–retest reliability, the two systems showed broadly similar reliability patterns. Displacement metrics were the most repeatable (median CV for pos_mag_ ≈ 3%), whereas velocity and acceleration metrics showed greater variability (median CV ≈ 20% and 40%, respectively). Although the study is exploratory with only three participants, the analysis of 42 tightly paired trials and more than 43,000 synchronized frames was sufficient to reveal performance patterns of a 25 Hz system at the algorithm-validation level.

Limitations and practical implications. This study has several limitations. First, the sample size was small (*n* = 3), and tasks were performed in a controlled laboratory setting without a ball, which may limit generalizability to game-like contexts involving contact, ball-handling, and multi-athlete occlusions. Second, the MMC workflow used six consumer-grade cameras to reflect field-deployment constraints; reduced camera redundancy may decrease multi-view keypoint visibility and triangulation robustness during high-speed, partially occluded actions, thereby disproportionately degrading numerically differentiated velocity and acceleration. Third, we used an out-of-the-box RTMPose model (COCO-style keypoints) without basketball-specific fine-tuning because task-specific labeled 2D keypoints or 3D ground truth were not available for supervised adaptation in the present dataset; domain adaptation may reduce keypoint jitter and improve derivative agreement. Fourth, higher acquisition rates (e.g., 50–100 Hz) were not evaluated, and the conservative 6 Hz low-pass filtering required at 25 Hz to suppress jitter inevitably attenuates high-frequency transients and may underestimate instantaneous derivative peaks. Taken together, these constraints clarify the intended positioning of this work in the trade-off between sampling rate and deployability: the 25 Hz setting represents an engineering compromise aligned with low-barrier training and sideline monitoring, where rapid feedback and feasible deployment often outweigh maximal precision for every instantaneous metric. Under these practical conditions, our findings delineate a “performance boundary” for consumer devices—displacement-dominant outputs can achieve high agreement, whereas velocity and acceleration derived by numerical differentiation remain the primary accuracy bottleneck. Future work should systematically quantify the benefits of higher frame rates under otherwise identical camera layout and processing, and, without substantially increasing deployment cost, prioritize improving synchronization accuracy and calibration stability, increasing camera redundancy where feasible, refining anatomical correspondence of keypoints, and exploring basketball-specific model adaptation and data augmentation, with validation extended to more ecologically complex settings involving multiple athletes, greater occlusion, and game-like contact and constraints.

## 5. Conclusions

This study validated the practical performance of an MMC workflow based on 25 Hz consumer-grade video streams for basketball-specific movements. The results demonstrate that, under the tested conditions requiring rapid feedback and non-instrumented data collection, this approach can provide accurate displacement-related 3D trajectories (r > 0.91) across joints and tasks. These displacement trajectories were analyzed as relative displacement magnitudes and can directly support displacement-based external-load descriptors such as distance covered, trajectory/path features, and mean speed derived from the 3D trajectories. We note that the present study did not evaluate higher-level biomechanical variables that require additional modeling assumptions, such as joint angles or center-of-mass metrics, which should be examined in dedicated follow-up work. In contrast, agreement for velocity and acceleration was substantially lower, reflecting an observed limitation under the current 25 Hz setting and processing pipeline, where micro timing/phase deviations and keypoint jitter introduced by markerless detection, tracking, and triangulation can be amplified by numerical differentiation. Because sampling rate was not manipulated in this study, isolating frame-rate effects will require future multi-rate experiments. Finally, given that the tasks were performed in a controlled laboratory environment without ball-handling or game-like interactions, we refrain from claiming ecological validity; instead, these findings delineate practical performance boundaries and support the workflow’s deployability potential for low-burden training monitoring, while field validation in more representative settings remains necessary. Future work should prioritize improving multi-camera temporal synchronization and incorporating basketball-specific pose priors to further mitigate the accuracy limitations of derivative variables.

## Figures and Tables

**Table 1:** Overall validity summary for displacement magnitude (pos_mag_) across 12 joints.

Joint	r (Mean ± SD)	RMSE (Mean ± SD)	nRMSE% (Median [Q1, Q3])	*n*
L Shoulder	0.994 ± 0.008	0.079 ± 0.106	0.59 [0.46, 1.23]	42
R Shoulder	0.993 ± 0.009	0.079 ± 0.110	0.63 [0.51, 1.13]	41
L Elbow	0.994 ± 0.009	0.087 ± 0.102	0.65 [0.40, 2.56]	42
R Elbow	0.994 ± 0.009	0.088 ± 0.118	0.92 [0.44, 1.17]	42
L Wrist	0.916 ± 0.201	0.176 ± 0.195	1.32 [0.82, 7.09]	42
R Wrist	0.959 ± 0.133	0.160 ± 0.303	0.57 [0.46, 0.99]	42
L Hip	0.989 ± 0.025	0.075 ± 0.105	0.74 [0.45, 0.98]	42
R Hip	0.990 ± 0.024	0.086 ± 0.106	0.81 [0.68, 1.46]	42
L Knee	0.992 ± 0.012	0.118 ± 0.153	0.63 [0.31, 3.95]	42
R Knee	0.994 ± 0.008	0.110 ± 0.136	0.72 [0.36, 3.77]	42
L Ankle	0.992 ± 0.014	0.081 ± 0.114	0.58 [0.24, 1.16]	42
R Ankle	0.989 ± 0.035	0.101 ± 0.167	0.54 [0.22, 1.85]	42

Note: Values are reported as r (mean ± SD), RMSE (mean ± SD), and nRMSE% (median [Q1, Q3]) for each joint.

**Table 2:** Overall validity summary for velocity magnitude (v_mag_) across 12 joints.

Joint	r (Mean ± SD)	RMSE (Mean ± SD)	nRMSE% (Median [Q1, Q3])	*n*
L Shoulder	0.841 ± 0.286	0.670 ± 1.439	3.06 [2.04, 4.53]	42
R Shoulder	0.867 ± 0.243	0.711 ± 1.550	3.32 [2.24, 4.14]	41
L Elbow	0.694 ± 0.321	0.955 ± 1.320	1.46 [0.82, 8.67]	42
R Elbow	0.640 ± 0.262	1.067 ± 1.454	3.21 [1.16, 5.50]	42
L Wrist	0.616 ± 0.370	1.285 ± 1.767	3.76 [0.70, 5.55]	42
R Wrist	0.799 ± 0.315	0.807 ± 1.483	2.04 [1.61, 2.97]	42
L Hip	0.712 ± 0.281	0.862 ± 1.421	1.69 [0.35, 3.95]	42
R Hip	0.583 ± 0.294	1.031 ± 1.453	2.74 [0.70, 5.42]	42
L Knee	0.612 ± 0.308	1.231 ± 1.551	2.15 [1.19, 10.10]	42
R Knee	0.658 ± 0.314	1.224 ± 1.553	2.55 [0.43, 10.22]	42
L Ankle	0.806 ± 0.313	0.838 ± 1.464	0.62 [0.44, 3.58]	42
R Ankle	0.836 ± 0.285	0.809 ± 1.596	0.70 [0.51, 1.87]	42

Note: Values are reported as r (mean ± SD), RMSE (mean ± SD), and nRMSE% (median [Q1, Q3]) for each joint.

**Table 3:** Overall validity summary for acceleration magnitude (a_mag_) across 12 joints.

Joint	r (Mean ± SD)	RMSE (Mean ± SD)	nRMSE% (Median [Q1, Q3])	*n*
L Shoulder	0.584 ± 0.238	14.294 ± 32.756	6.75 [4.31, 8.31]	42
R Shoulder	0.573 ± 0.204	15.600 ± 35.448	8.45 [5.54, 10.92]	41
L Elbow	0.346 ± 0.304	23.986 ± 30.738	1.70 [0.43, 7.80]	42
R Elbow	0.257 ± 0.277	27.185 ± 32.712	3.05 [1.30, 5.92]	42
L Wrist	0.386 ± 0.329	31.906 ± 44.454	2.97 [0.33, 4.56]	42
R Wrist	0.557 ± 0.301	16.715 ± 33.964	3.16 [2.35, 4.82]	42
L Hip	0.346 ± 0.264	20.082 ± 32.931	1.07 [0.20, 4.31]	42
R Hip	0.232 ± 0.279	25.378 ± 33.108	2.85 [0.28, 4.61]	42
L Knee	0.292 ± 0.289	30.656 ± 36.366	2.08 [1.15, 8.16]	42
R Knee	0.388 ± 0.294	30.330 ± 37.041	2.86 [0.25, 9.09]	42
L Ankle	0.615 ± 0.369	19.171 ± 34.315	0.30 [0.22, 3.58]	42
R Ankle	0.677 ± 0.300	17.219 ± 37.350	0.30 [0.20, 0.84]	42

Note: Values are reported as r (mean ± SD), RMSE (mean ± SD), and nRMSE% (median [Q1, Q3]) for each joint.

**Table 4:** Waveform validity results under different task conditions.

Task ID	Task Name	pos_mag_ *r*_mean_ (pos_mag_)	RMSE (pos_mag_, m)	*r*_mean_ (vmag)	RMSE (v_mag_, m/s)	*r*_mean_ (a_mag_)	RMSE (a_mag_, m/s^2^)
Tri01	Lane Drill Walk	0.999	0.035	0.784	0.294	0.404	7.633
Tri02	Lane Drill Run	0.999	0.039	0.800	0.357	0.434	9.425
Tri03	Lane Drill Sprint	0.996	0.086	0.778	0.704	0.402	17.902
Tri04	Drop-step vertical jump	0.998	0.029	0.824	0.389	0.574	10.321
Tri05	Free-throw simulation	0.939	0.045	0.866	0.199	0.593	5.228
Tri06	Layup (3-step)	0.978	0.366	0.181	4.423	0.061	102.351
Tri07	Crossover + step-back jump shot	0.972	0.124	0.820	0.331	0.595	6.002

**Table 5:** Repeated-measures Bland–Altman summary of frame-level agreement between MMC and Vicon across 12 joints. Bias indicates the mean MMC–Vicon difference; LoA denotes the 95% limits of agreement. N represents pooled joint–frame observations (paired values at each frame for each joint) after excluding one trial with incomplete joint coordinates.

Variable	Bias	LoA (Lower)	LoA (Upper)	N (Joint–Frame Points)	Subjects
pos_mag_ (m)	0.0029	−0.4252	0.4310	510,540	3
v_mag_ (m/s)	−0.0163	−6.3245	6.2919	510,048	3
a_mag_ (m/s^2^)	−1.0451	−194.4521	192.3620	509,556	3

Note: N represents joint–frame observations pooled across 12 joints and all valid frames.

**Table 6:** Test–retest reliability summary (mean feature) for MMC and Vicon across 12 joints and 7 tasks.

System	Variable	ICC(A,1) (Median, Range)	CV% (Median, Range)	MDC95 (Median)
MMC	pos_mag_	0.00 (−2.01–1.00)	3.97 (0.00–39.22)	0.111
MMC	v_mag_	0.52 (−0.25–0.99)	3.93 (0.09–16.45)	0.073
MMC	a_mag_	0.44 (−0.34–0.99)	6.22 (0.00–23.07)	0.504
Vicon	pos_mag_	0.29 (−0.33–1.00)	4.00 (0.00–39.23)	0.113
Vicon	v_mag_	0.37 (−0.20–0.99)	4.52 (0.05–15.85)	0.075
Vicon	a_mag_	0.28 (−0.31–0.99)	12.99 (0.00–51.05)	0.787

## Data Availability

Due to privacy and ethical restrictions, the raw multi-view video recordings collected for this study are not publicly available. The processed kinematic datasets exported from the reference optical motion-capture system (Vicon) and the markerless motion-capture pipeline (MMC) (i.e., 3D joint-center trajectories in the global coordinate system) will be made publicly available in an open repository upon acceptance of this manuscript; prior to that, these data are available from the corresponding authors upon reasonable request. The source code of the MMC pipeline cannot be shared publicly because it contains proprietary components protected by intellectual property/patent restrictions. Key processing parameters and outcome-variable definitions are provided in the manuscript and Supplementary Materials.

## Supplementary Materials

The following supporting information can be downloaded at: https://www.mdpi.com/article/10.3390/s26051689/s1. SA. Supplementary Methods; SB. Supplementary Figures. Figure S1. Waveforms across 12 joints for velocity magnitude (v_mag_). (a) Full-scale visualization preserving extreme transient peaks. (b) Zoomed-in visualization where y-axis limits were set for each joint separately to the 1st–99th percentiles, computed from values pooled across MMC and Vicon within that joint, to highlight the bulk waveform patterns. Figure S2. Waveforms across 12 joints for acceleration magnitude (a_mag_). (a) Full-scale visualization preserving extreme transient peaks. (b) Zoomed-in visualization where y-axis limits were set for each joint separately to the 1st–99th percentiles, computed from values pooled across MMC and Vicon within that joint, to highlight the bulk waveform patterns. Figure S3. Pooled Bland–Altman plot of frame-level agreement between MMC and Vicon for velocity magnitude (v_mag_) across 12 joints (25 Hz; 6 Hz filtering). The center line denotes the mean bias and dashed lines denote the 95% limits of agreement. Figure S4. Pooled Bland–Altman plot of frame-level agreement between MMC and Vicon for acceleration magnitude (a_mag_) across 12 joints (25 Hz; 6 Hz filtering). The center line denotes the mean bias and dashed lines denote the 95% limits of agreement. Figure S5. Task-level waveform validity across seven basketball-specific tasks. Figure S6. Joint-wise distributions of waveform correlation (*r*) across all paired trials for displacement (pos_mag_), velocity (v_mag_), and acceleration (a_mag_) magnitudes. Boxplots summarize the median and interquartile range, with whiskers indicating dispersion across trials. Figure S7. Power spectral density (PSD) of displacement magnitude (pos_mag_), velocity magnitude (v_mag_), and acceleration magnitude (a_mag_) for MMC and Vicon in a representative paired trial, shown before (raw) and after 6 Hz zero-phase low-pass filtering (LPF). PSD curves are summarized as the median across the 12 joints; SC. Supplementary Tables. Table S1. Sensitivity analysis of task-level waveform validity estimates with and without Tri06 (Layup, 3-step). Table S2. Frame-level concordance between MMC and Vicon across 12 joints (pooled joint–frame points), reporting Pearson’s r, Lin’s concordance correlation coefficient (CCC), and mean difference (MMC–Vicon). Table S3. Summary of test–retest reliability for the peak feature (pooled across 12 joints × 7 tasks), including ICC(A,1), CV%, and MDC95. Table S4. Summary of test–retest reliability for the P95 feature (pooled across 12 joints × 7 tasks), including ICC(A,1), CV%, and MDC95. Table S5. Fraction of total power spectral density (PSD) below 6 Hz for displacement, velocity, and acceleration magnitudes (median across 12 joints) for MMC and Vicon, before and after 6 Hz low-pass filtering.
