# Supplementary material for: Evaluating a Multi-Camera Markerless System for Capturing Basketball-Specific Movements: An Exploration Using 25 Hz Video Streams
Source: Sensors (Basel). 2026 Mar 7;26(5):1689. doi: 10.3390/s26051689 (PMC12986566; doi:10.3390/s26051689)
Supplement: Supplementary file 1 [file sensors-26-01689-s001.zip › sensors-4153859-supplementary.pdf]

# Evaluating a Multi-Camera Markerless System for Capturing Basketball-Specific Movements: An Exploration Using 25 Hz Video Streams

Supplementary Material

Contents

A. SUPPLEMENTARY METHODS ........ 1
B. SUPPLEMENTARY FIGURES ........ 2
C. SUPPLEMENTARY TABLES ........ 7

## A. Supplementary Methods

### A.1 Representative-Trial Selection for Waveform Plots

One paired trial was visualized to provide an intuitive illustration of temporal agreement between MMC and Vicon. This trial was selected a priori as a representative (non-outlier) case based on the distribution of trial-level validity metrics (e.g., median-level r and RMSE across joints) and the absence of missing axes. Importantly, all inferential statements in the main text are supported by the full set of paired trials and summarized quantitatively in Tables 2–5 and Supplementary Figures S1–S6 and Tables S1-S4.

### A.2 Additional Quality Control and Exclusion Rules

Paired trials were identified by parsing subject/session/task identifiers from file names and matching MMC and Vicon files on a 1:1 basis. Frame-level alignment was performed using the synchronized event and a shared frame_index. Trials with missing joint-axis components were retained for analyses not involving the affected joint, but were excluded from frame-level agreement analyses requiring complete 12-joint data. Specifically, one paired trial had incomplete right-shoulder z coordinates in both systems; thus, right-shoulder trial-level summaries used $n = 41$, and this trial was excluded from pooled frame-level Bland–Altman analyses.

### A.3 Signal Processing Details

All 3D joint trajectories were sampled at 25 Hz. To attenuate reconstruction jitter and reduce noise amplification in numerical differentiation, each axis component was low-pass filtered using a 4th-order Butterworth filter with a 6 Hz cutoff and zero-phase forward–backward filtering (filtfilt). Velocity and acceleration were then computed using central differences on the filtered displacement signals. Frequency-domain evidence is provided in Supplementary Fig. S7 and Table S5.

### A.4 Frequency-domain evidence supporting the 6 Hz cutoff

To justify the conservative 6 Hz low-pass cutoff used at 25 Hz, we inspected the

frequency content of the magnitude signals (pos_mag, v_mag, a_mag). Power spectral density (PSD) was estimated for MMC and Vicon and summarized as the median PSD across the 12 joints. As shown in Supplementary Fig. S7, displacement and velocity magnitudes exhibited predominantly low-frequency content, with nearly all power concentrated below 6 Hz. Acceleration magnitude contained relatively more high-frequency energy—particularly for MMC—consistent with increased sensitivity of second derivatives to keypoint jitter and subtle timing offsets. Supplementary Table S5 reports the fraction of signal power below 6 Hz, indicating that the selected cutoff preserves the bulk of the signal while suppressing high-frequency jitter that would otherwise be amplified by numerical differentiation.

## B. Supplementary Figures

### B.1 Waveform Visualization

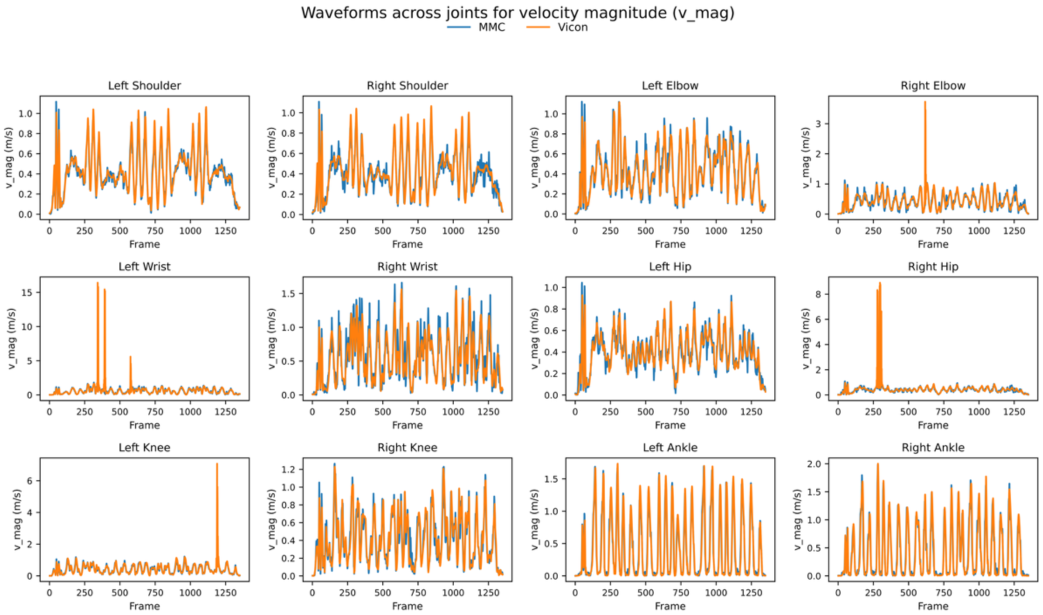

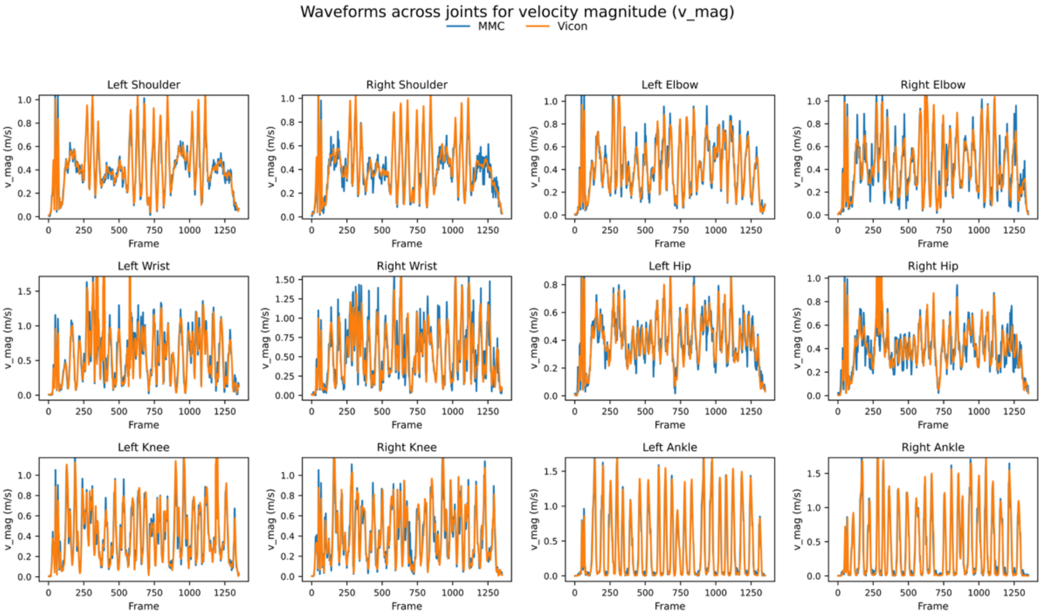


**Figure S1.** Waveforms across 12 joints for velocity magnitude (v_mag). (a) Full-scale visualization preserving extreme transient peaks. (b) Zoomed-in visualization where y-axis limits were set for each joint separately to the 1st–99th percentiles, computed from values pooled across MMC and Vicon within that joint, to highlight the bulk waveform patterns.

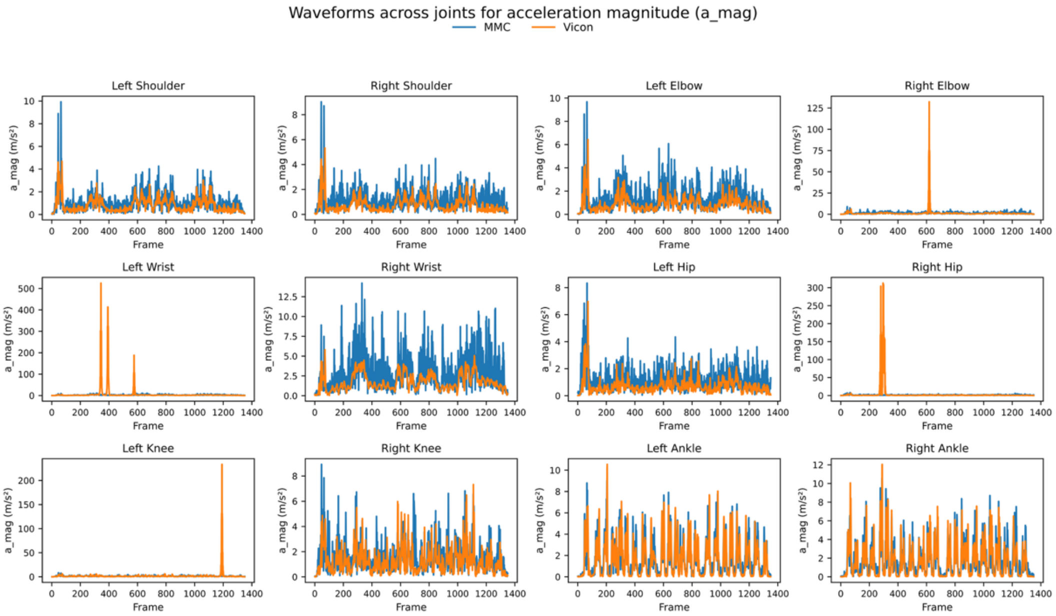

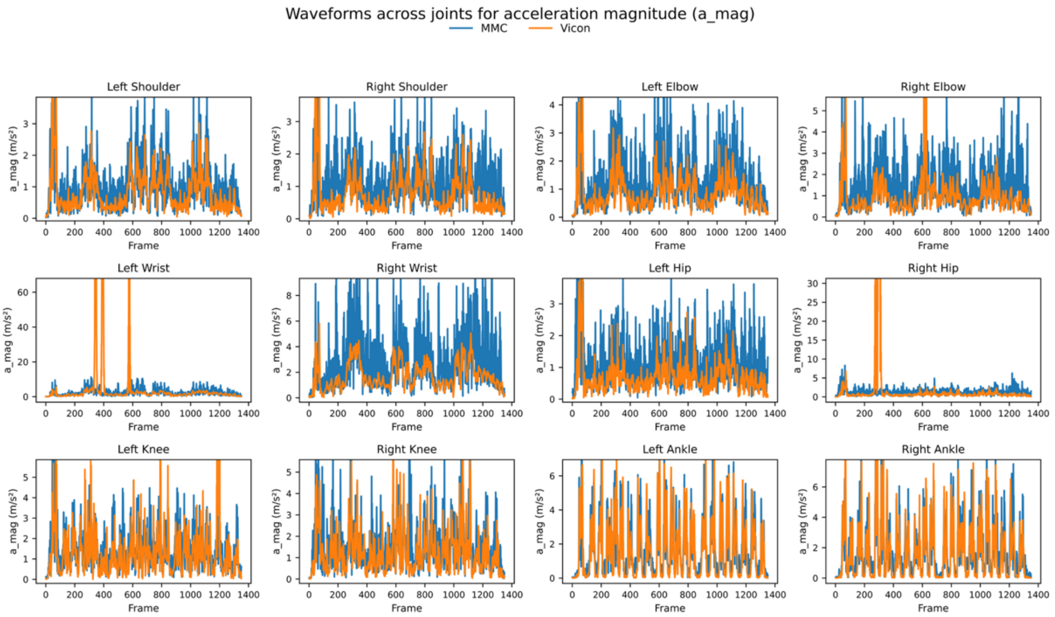


**Figure S2.** Waveforms across 12 joints for acceleration magnitude (a_mag). (a) Full-scale visualization preserving extreme transient peaks. (b) Zoomed-in visualization where y-axis limits were set for each joint separately to the 1st–99th percentiles, computed from values pooled across MMC and Vicon within that joint, to highlight the bulk waveform patterns.

## B.2 Frame-Level Agreement Plots

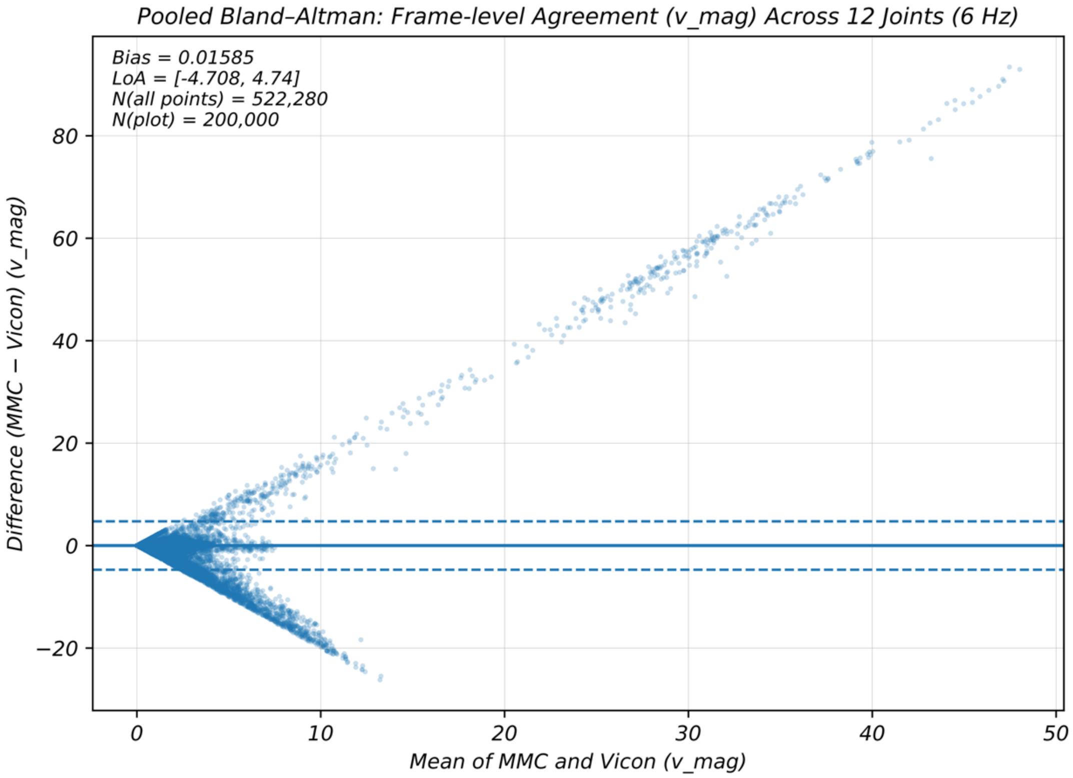

**Figure S3.** Pooled Bland–Altman plot of frame-level agreement between MMC and Vicon for velocity magnitude (v_mag) across 12 joints (25 Hz; 6 Hz filtering). The center line denotes the mean bias and dashed lines denote the 95% limits of agreement.

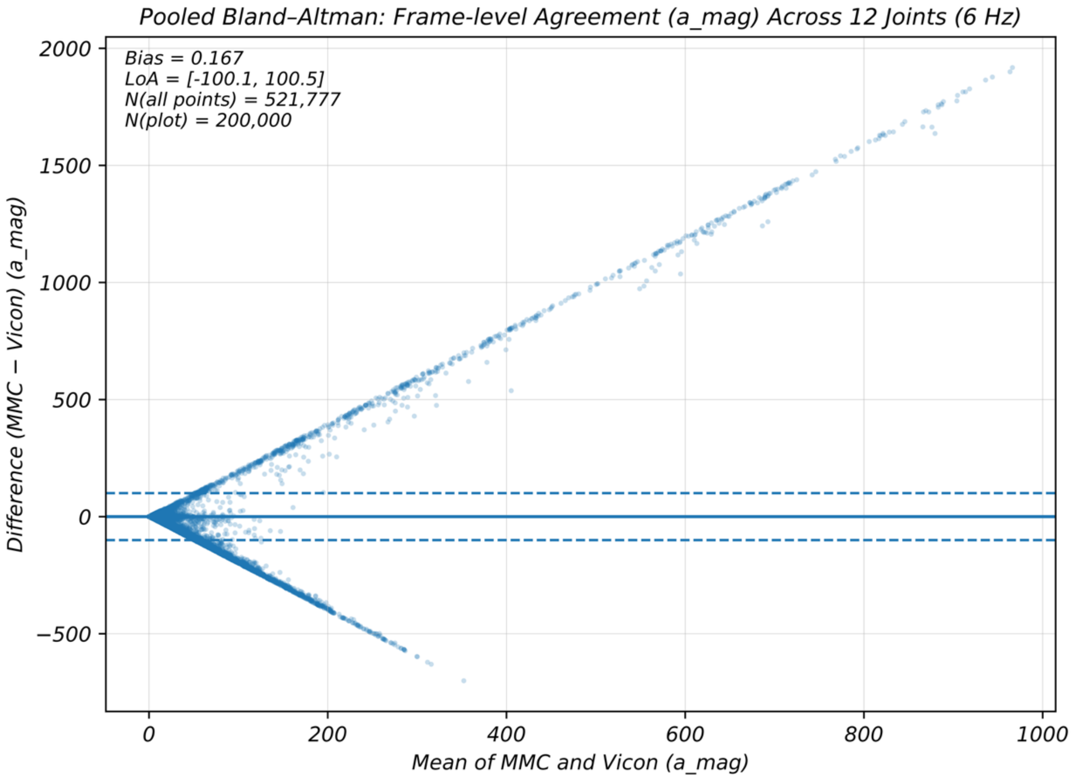


**Figure S4.** Pooled Bland–Altman plot of frame-level agreement between MMC and Vicon for acceleration magnitude (a_mag) across 12 joints (25 Hz; 6 Hz filtering). The center line denotes the mean bias and dashed lines denote the 95% limits of agreement.

**B.3 Distribution and Robustness Evidence**

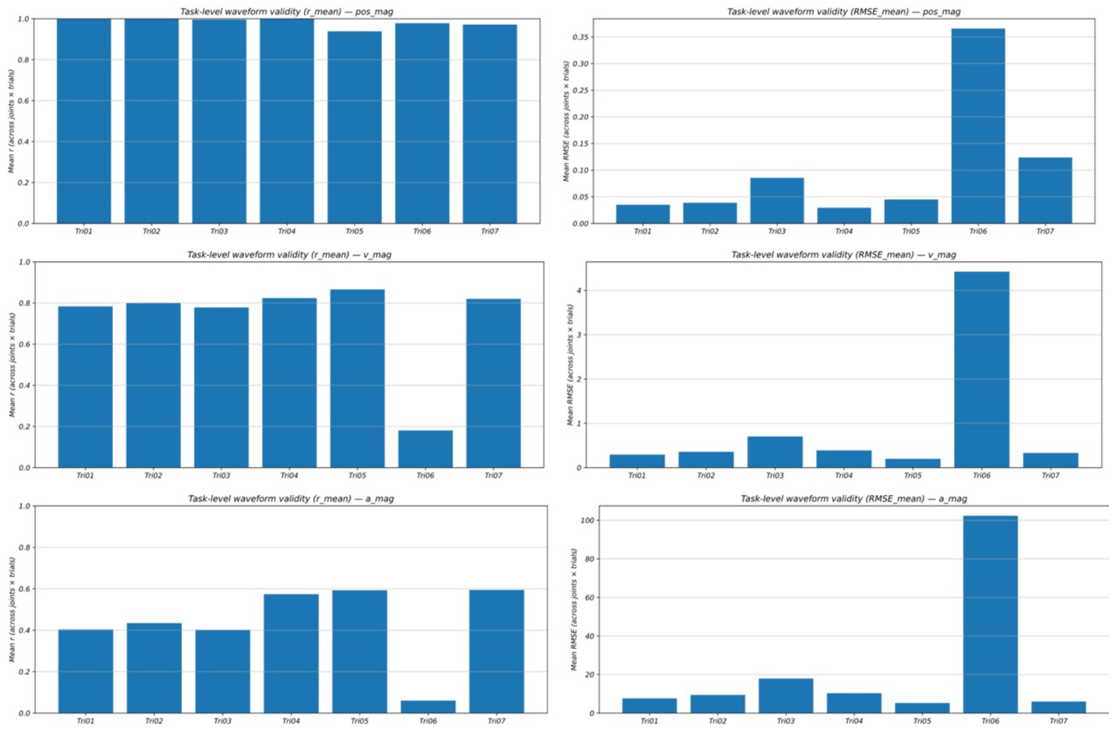


**Figure S5.** Task-level waveform validity across seven basketball-specific tasks.

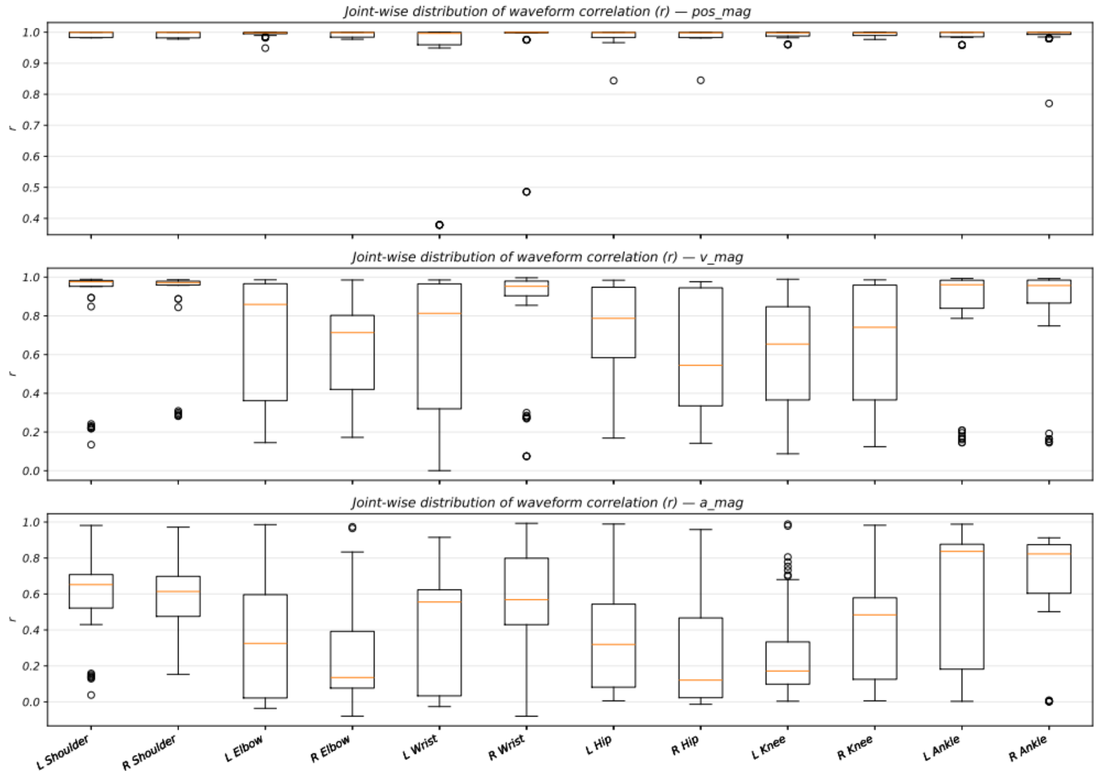


**Figure S6.** Joint-wise distributions of waveform correlation (r) across all paired trials for displacement (pos_mag), velocity (v_mag), and acceleration (a_mag) magnitudes. Boxplots summarize the median and interquartile range, with whiskers indicating dispersion across trials.

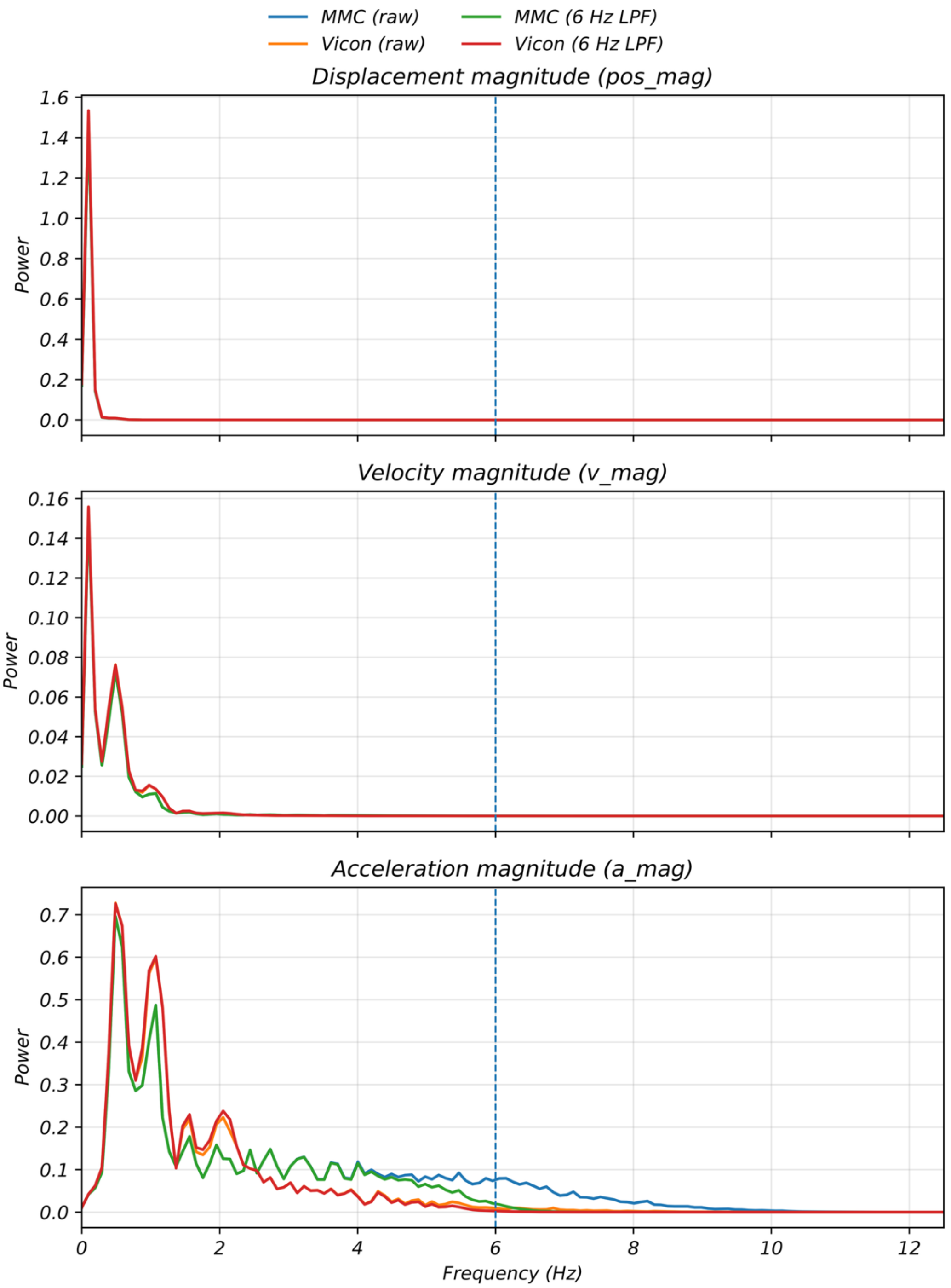


**Figure S7.** Power spectral density (PSD) of displacement magnitude (pos_mag), velocity magnitude (v_mag), and acceleration magnitude (a_mag) for MMC and Vicon in a representative paired trial, shown before (raw) and after 6 Hz zero-phase low-pass filtering (LPF). PSD curves are summarized as the median across the 12 joints.

## C. Supplementary Tables

**Table S1.** Sensitivity analysis of task-level waveform validity estimates with and without Tri06 (Layup, 3-step).

| Variable | Task-level mean r_mean (All tasks, n=7) | Task-level mean r_mean (Excluding Tri06, n=6) | Δr | Task-level mean RMSE_mean (All tasks) | Task-level mean RMSE_mean (Excluding Tri06) | ΔRMSE | RMSE change |
|---|---|---|---|---|---|---|---|
| pos_mag (m) | 0.983 | 0.984 | +0.001 | 0.103 | 0.060 | −0.044 | −42.3% |
| v_mag (m/s) | 0.722 | 0.812 | +0.090 | 0.957 | 0.379 | −0.578 | −60.4% |
| a_mag ($m/s^2$) | 0.437 | 0.500 | +0.063 | 22.694 | 9.418 | −13.276 | −58.5% |

**Notes:**
*(1) Task-level metrics (r_mean, RMSE_mean) were defined as in Table 3, i.e., averaged across joints and trials within each task. "All tasks" summarizes the mean across the 7 movement tasks (Tri01–Tri07).*
*(2) Tri06 (Layup, 3-step) was identified as an influential task because it showed simultaneously low task-level correlation and high task-level RMSE for derivative variables (v_mag and a_mag) relative to the across-task distribution. To quantify its influence on pooled task-level estimates, we recomputed the across-task means after excluding Tri06.*
*(3) Excluding Tri06 substantially reduced pooled task-level RMSE for v_mag and a_mag (≈58–60% reduction) and increased pooled task-level r_mean, indicating that derivative-variable agreement estimates are sensitive to this high-displacement, complex-posture task. The primary conclusions of the study remain unchanged (pos_mag shows the highest agreement, derivative variables exhibit larger errors), but derivative-variable results for Tri06 should be interpreted conservatively.*

**C.1 Sensitivity Analysis for Tri06 (Layup, 3-step)**

To evaluate whether the task-level pooled validity estimates were unduly influenced by a single movement condition, we performed a sensitivity analysis focusing on Tri06 (Layup, 3-step). Tri06 exhibited the weakest derivative-variable agreement in the main results (Table 3), characterized by markedly reduced task-level waveform correlation (r_mean) and substantially inflated task-level error (RMSE_mean) for both v_mag and a_mag. Because derivative variables amplify small timing misalignments and reconstruction jitter, high-displacement and complex-posture tasks may act as influential conditions in pooled summaries.

Following a standard exploratory screening approach, Tri06 was treated as an influential task at the task level because it simultaneously presented low correlation and high RMSE compared with the across-task distribution (7 tasks). We therefore recomputed pooled task-level means across tasks for each magnitude variable using (i) all tasks (Tri01–Tri07) and (ii) excluding Tri06 only. Results are reported in Table S1.

Excluding Tri06 increased the pooled task-level correlation for v_mag (0.722 to 0.812) and a_mag (0.437 to 0.500), while markedly reducing pooled task-level RMSE (v_mag: 0.957 to 0.379 m/s; a_mag: 22.694 to 9.418 $m/s^2$). The displacement magnitude (pos_mag) remained

highly correlated regardless of inclusion, with a moderate reduction in pooled RMSE (0.103 to 0.060 m). Collectively, these findings indicate that derivative-variable validity estimates are sensitive to Tri06, whereas the overarching conclusion—high agreement for displacement and larger uncertainty for derivative variables—remains robust. Consequently, Tri06-specific derivative metrics should be interpreted conservatively, and reporting both full-task and sensitivity summaries improves transparency for applied deployment scenarios.

**C.2 Concordance Correlation Coefficient (CCC)**

To complement the Bland–Altman agreement analysis, Lin's concordance correlation coefficient (CCC) was computed to quantify the overall concordance between MMC and the reference system (Vicon) at the frame level. CCC evaluates both precision (correlation) and accuracy (deviation from the identity line). Frame-level paired observations were pooled across the 12 joints and all valid frames/trials (consistent with the repeated-measures Bland–Altman dataset used in Table 4 of the main text). Table S2

summarizes CCC and Pearson's r for the three magnitude variables.

**Table S2.** Frame-level concordance between MMC and Vicon across 12 joints (pooled joint-frame points).

| Variable | N (joint-frame points) | Pearson r | Lin's CCC | Mean difference (MMC−Vicon) |
|---|---|---|---|---|
| pos_mag | 510,540 | 0.988 | 0.987 | 0.0029 |
| v_mag | 510,048 | 0.785 | 0.537 | -0.0163 |
| a_mag | 509,556 | 0.701 | 0.477 | -1.0451 |

**C.3 Test–retest reliability of P95 and peak features**

In addition to the mean feature reported in the main text, test–retest reliability was also evaluated for peak and 95th percentile (P95) features extracted from the magnitude time series (pos_mag, v_mag, a_mag). For each system and each trial, peak and P95 were computed from the filtered magnitude series, and reliability between Day 1 and Day 2 was assessed using ICC(A,1), CV%, and MDC95 (definitions consistent with the main text). To maintain concision in the main manuscript, only mean-feature summaries were included there; the peak and P95 summaries are provided here (Tables S3–S4).

**Table S3.** Summary of test–retest reliability for the peak feature (pooled across 12 joints × 7 tasks).

| System | Variable | ICC(A,1) (median, range) | CV% (median, range) | MDC95 (median) |
|---|---|---|---|---|
| MMC | pos_mag | 0.03 (-3.25–1.00) | 2.92 (0.00–26.22) | 0.133 |
| MMC | v_mag | 0.17 (-1.17–0.98) | 4.66 (0.20–18.41) | 0.361 |
| MMC | a_mag | 0.16 (-1.85–1.00) | 7.05 (1.33–76.81) | 3.935 |
| Vicon | pos_mag | 0.01 (-2.36–1.00) | 2.92 (0.00–31.87) | 0.186 |
| Vicon | v_mag | 0.13 (-1.09–1.00) | 9.64 (0.50–141.34) | 1.122 |
| Vicon | a_mag | 0.17 (-0.96–1.00) | 15.63 (0.26–208.50) | 33.230 |

**Table S4.** Summary of test–retest reliability for the P95 feature (pooled across 12 joints × 7 tasks).

| System | Variable | ICC(A,1) (median, range) | CV% (median, range) | MDC95 (median) |
|---|---|---|---|---|
| MMC | pos_mag | 0.03 (-9.85–1.00) | 3.04 (0.00–35.49) | 0.112 |
| MMC | v_mag | 0.36 (-0.88–0.98) | 4.90 (0.39–34.44) | 0.201 |
| MMC | a_mag | 0.38 (-1.48–1.00) | 7.28 (1.23–42.03) | 1.453 |
| Vicon | pos_mag | 0.02 (-1.43–1.00) | 2.64 (0.00–37.55) | 0.108 |
| Vicon | v_mag | 0.39 (-1.42–1.00) | 5.25 (1.00–81.80) | 0.221 |
| Vicon | a_mag | 0.33 (-1.49–1.00) | 13.72 (0.47–200.19) | 2.151 |

**Table S5.** Fraction of total PSD power below 6 Hz for pos_mag, v_mag, and a_mag (median across 12 joints) for MMC and Vicon, before and after 6 Hz low-pass filtering.

| variable | stage | $MMC_{power} \leqslant 6Hz$ | $Vicon_{power} \leqslant 6Hz$ |
|---|---|---|---|
| pos | raw | 0.999997 | 1.000000 |
| pos | filt | 1.000000 | 1.000000 |
| v | raw | 0.995275 | 0.999552 |
| v | filt | 0.999752 | 0.999973 |
| a | raw | 0.874327 | 0.983667 |
| a | filt | 0.990742 | 0.998902 |
